# The Rewards and Challenges of Interdisciplinary Collaborations

**DOI:** 10.1016/j.isci.2019.08.015

**Published:** 2019-08-22

**Authors:** Z. Jason Ren

## Main Text

**Figure:**
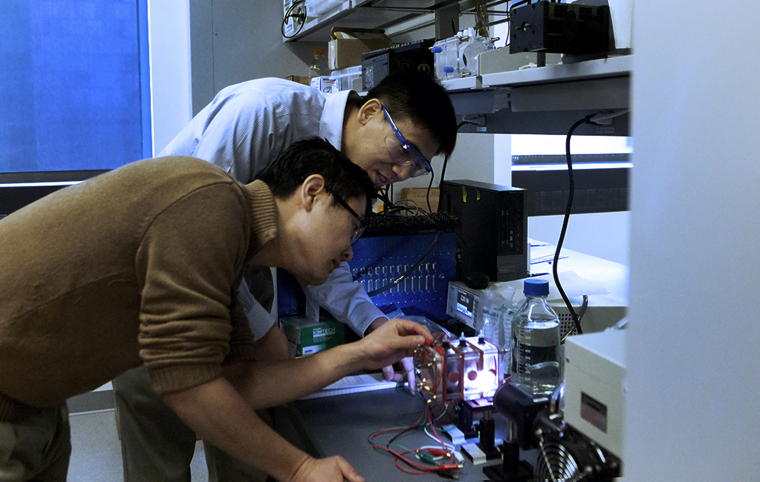
Jason Ren and Lu Lu (engineers) and Jing Gu (chemist) developed a new method to separate hydrogen from wastewater with record performance (Photo by Bumper DeJesus, Princeton).

Insight from, and dialog between, multiple scientific disciplines is accepted as a necessity when trying to understand complex scientific questions. An important prerequisite of finding answers to these queries, however, is the ability to effectively organize and manage diverse research teams. Zhiyong Jason Ren (@zjasonren), an environmental engineer and interdisciplinary project lead guru, has learned that it takes courage, convergence, and common goals to find success.

Ren, who received his PhD in environmental engineering from Penn State University, now is the Associate Director for Research at the interdisciplinary Andlinger Center for Energy and the Environment at Princeton University. He also directs the Princeton Water & Energy Technologies (WET) Laboratory in the Department of Civil and Environmental Engineering. Currently, his research focuses on water-energy nexus and resource recovery from wastewater, placing him at the intersection of the fields of microbiology, energy, material science, and engineering.

Below Ren details his experiences working in interdisciplinary research groups—what works, what doesn't, and what steps others can take to join in this rewarding venture.

## Proximity

### Interdisciplinary Collaborations Require Stepping Outside of Comfort Zones

Proximity is a big catalyst for interdisciplinary collaboration, but it is not always necessary. It's likely that a project will begin with a nearby colleague, but you will find that it can continue uninterrupted despite relocation to more distant institutions. For example, I worked on a proposal with a collaborator when we were both living in Colorado, but now I'm on the east coast and they're on the west coast, and our work is completely intact—our ability to collaborate hasn't been hindered at all.

There are also times when collaborations begin from afar as well, especially with large, multi-million-dollar proposals, which can require you to actively search for the right people to work with. And just because you're choosing who you're reaching out to, it doesn't necessarily mean that you'll be working with your friends because ultimately, they might not have the expertise you're looking for. *However, that's one of the best parts of interdisciplinary research—it forces you to step out of your comfort zone and seek new collaborators. And I am always surprised by what I find.*

But that's one of the best parts of interdisciplinary research – it forces you to step out of your comfort zone and seek new collaborators. And I am always surprised by what I find.

## Language

### Have Conversations to Identify Common Ground

**Figure:**
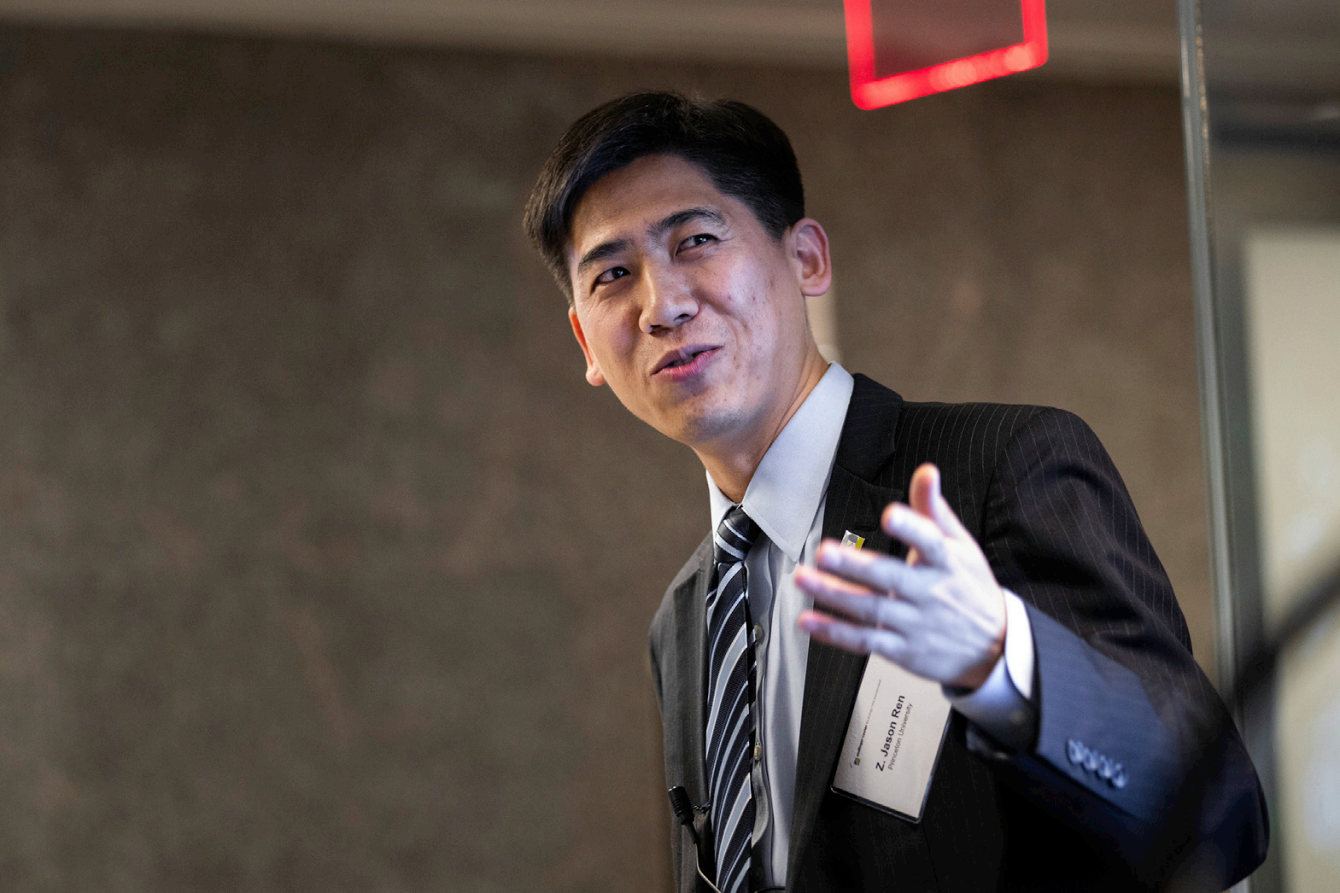
Prof. Ren was giving a talk on water-energy nexus at an interdisciplinary workshop at the Andlinger Center for Energy and the Environment at Princeton (Photograph by Tori Repp, Fotobuddy).

In the beginning you can likely anticipate two main issues: confusion in terms of the jargon other disciplines use and disagreement about the focus of the project. Everybody will be approaching the same topic but with vastly different opinions, expertise, and personalities. For example, one camp of researchers might be concerned with expanding the fundamental understanding of a topic, whereas the other camp might be more interested in figuring out how to apply their research into design and technology development.

So there certainly might be some clashes, but I've found that one way to overcome this is to find common ground and emphasize synergy and collaboration over competition.

So there certainly might be some clashes, but I've found one way to overcome this is to find common ground and emphasize synergy and collaboration over competition.

## Research Methods

### Let People Work to Their Strengths

I truly appreciate the interdisciplinary nature of the research in my laboratory. Currently, I have engineers, material scientists, chemists, and also biologists. They all work together and share a common goal, but simultaneously, they are working on aspects of the project where their strengths are most pronounced.

When sharing a common goal on doing and publishing the best research, the advantage of interdisciplinary team becomes obvious. People with complementary expertise often brainstorm out the best ideas, and members can co-author multiple articles with different focuses.

## Governance

### Opportunities and Challenges in Interdisciplinary Research

I consider appealing to grant organizations both a challenge and an opportunity, depending on the funding agency.

I consider appealing to grant organizations both a challenge and an opportunity, depending on the funding agency.

There is a new word being used in many largest funding opportunities for interdisciplinary research at the US National Science Foundation: convergence. Convergence means—and mandates—that big proposals pull people from a variety of disciplines, not only from the physical and life sciences but also from social sciences like economics and political science. The reason for this is that, of course, no single discipline can solve the big challenges alone.

The proposal is hard to write and to win, but it can be fun and informative as well. It's an opportunity to learn from the other disciplines involved in the project. I've had experiences where it has been a very efficient process—where we write the proposal together yet receive the funding separately and independently.

The proposal is hard to write and to win, but it can be fun and informative as well. It's an opportunity to learn from the other disciplines involved on the project.

However, it can be much more challenging to get funding for interdisciplinary work from mission-driven agencies. This is largely because the organization is unable to justify the broad reach of the research with the often defined scope of their missions.

Ultimately, it may take some tweaking of your proposal, but sooner or later you can find a good match for your interdisciplinary research.

## Publication

### As More Journals Support Interdisciplinary Research, More Authors Conduct It

**Figure:**
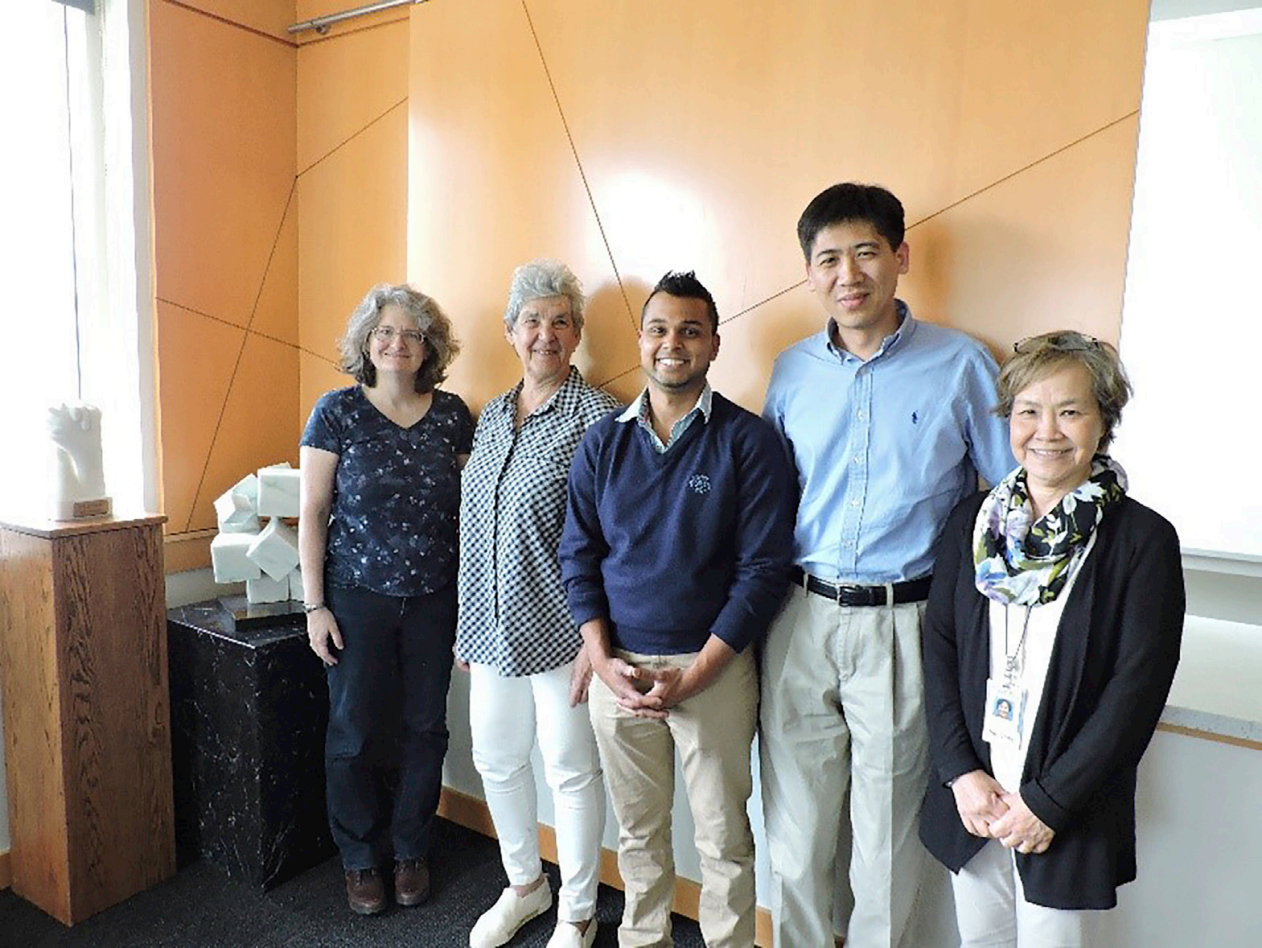
Prof. Ren's student Joshua Jack (center) with his committee members (from left: Profs. Angela Bielefeldt, JoAnn Silverstein, Jason Ren, and Pin-Ching Maness). Joshua successfully defended his PhD thesis in environmental engineering after working for 2 years in a microbiology laboratory led by Dr. Maness at the National Renewable Energy Laboratory. (Photograph by Laurence Lambert, University of Colorado Boulder).

There are big pictures that you need to communicate to all the different research communities, which is one of the most important aspects of interdisciplinary research. But then, there is also a need for in-depth methods and results descriptions that may be field specific. To address this, I spend a lot of time revising the introduction and discussion to ensure that those big-picture opportunities are center stage, and then the other sections can be divided into subcategories, with each person in the team writing more narrowly about their focus in the project.

Emphasizing the overarching scope of the project can create challenges for publishing in specific fields, but conversely, it can also be helpful because there are currently a lot of journals that are actively encouraging interdisciplinary research. Journals like *iScience* are doing a phenomenal job because you're established in a way where you have a diverse pool of editors and reviewers who will appreciate the broader scope of an article. This is really important to a researcher because two of our main considerations are which paper is publishing in what journal and from where are we getting funding to enable this research? If there is a strong pull from the publication side, and a push from the funding side, it will drive researchers to take the interdisciplinary route.

Journals like *iScience* are doing a phenomenal job because you're established in a way where you have a diverse pool of editors and reviewers who will appreciate the broader scope of an article.

## Conclusion

### Final Thoughts

Although navigating interdisciplinary collaborations can be challenging, ultimately, you'll find a common goal and deliver great outcomes. If you're unsure how to get started, I recommend stepping outside of your laboratory. Talk with people. Keep your mind open. Don't be shy. If you don't know their jargon, don't worry—they don't know yours either. I can promise you one thing, at the end of the day you feel you learnt something new that you got smarter.

The most impactful articles not only have deep science but also tell a compelling story—they tell why the science is important. The variety of perspectives provided by interdisciplinary research is vast, and results in fascinating science.

